# Attenuated *Salmonella typhimurium* L forms suppress tumor growth and promote apoptosis in murine ovarian tumors

**DOI:** 10.1038/s41598-024-66898-x

**Published:** 2024-07-11

**Authors:** Yunjie Zhang, Ziqing Tang, Yidan Shao, Xiaoli Yue, Yifan Chu, Dengyu Chen

**Affiliations:** 1 Department of Microbiology, Bengbu Medical University, Bengbu, 233030 Anhui People’s Republic of China; 2 Anhui Key Laboratory of Infection and Immunity, Bengbu Medical University, Bengbu, 233030 Anhui People’s Republic of China; 3 Department of Ophthalmology, The First Affiliated Hospital of Bengbu Medical University, Bengbu, 233000 Anhui People’s Republic of China; 4 Laboratory Center for Morphology, Bengbu Medical University, Bengbu, 233030 Anhui People’s Republic of China

**Keywords:** *Salmonella typhimurium*, *ST*, L forms, Epithelial ovarian cancer, Tumorigenicity, Apoptosis, Biological techniques, Biotechnology, Cancer, Cell biology, Drug discovery, Immunology, Microbiology, Medical research, Oncology, Pathogenesis

## Abstract

To study the effects of attenuated *Salmonella typhimurium* L forms on the in vivo tumorigenicity and apoptosis of murine epithelial ovarian cancer cells, as well as the related mechanisms. Attenuated *Salmonella typhimurium* VNP20009 was induced into bacterial L forms by using antibiotic ceftriaxone. CCK-8 cell proliferation assay showed that attenuated *S. typhimurium* L forms can inhibit the proliferation of murine ovarian epithelial cancer ID8 cells. Attenuated *ST* L forms can induce apoptosis and inhibit invasion ability of epithelial ovarian cancer cells in vitro. TUNEL assay showed that attenuated *ST* L forms can induce apoptosis of ID8 cells in murine ovarian tumors. Meanwhile, attenuated *ST* L forms inhibit tumor growth in murine ovarian tumors. The tumorigenicity-related proteins of xenograft tumors detected by immunohistochemistry and fluorescence quantitative RT-PCR assays showed that attenuated *ST* L forms can reduce the expression of proteins that promote tumor growth and metastasis, such as Lgals9 and MMP9. This study confirmed that attenuated *ST* L forms can suppress tumor growth and promote apoptosis in murine ovarian tumors. Attenuated *ST* L forms may serve as a novel biological agent for bacterial-mediated tumor therapy in epithelial ovarian cancer.

## Introduction

*Salmonella typhimurium* (*S. typhimurium*; *ST*) is one gram-negative bacillus and belongs to the facultative anaerobe of Enterobacteriaceae. *S. typhimurium* is one of the important zoonotic pathogens. Its incidence rate of infection ranks first in *Salmonella* infection, which is mostly seen in acute infant gastroenteritis and septicemia^1^. In recent years, research has found that *Salmonella typhimurium* is more inclined to aggregate in cancer cell spaces in malignant tumor tissues than other bacteria, inducing antitumor immunity, and inhibiting tumor growth and metastasis^2^. Modified and attenuated *Salmonella typhimurium* can be used for molecular delivery of antitumor drugs in cancer treatment, which can better control tumor growth and metastasis^3,4^. It is one of the cost-effective candidates for antitumor biological preparations. But currently, the clinical practical effect is not ideal, and further optimization and improvement are needed. This bacterium should been modified better and attenuated for potential therapeutic applications.

Epithelial ovarian cancer (EOC) occurs in the ovary. The location of the tumor is hidden. It is often found in the middle and late stages. After surgery, it also often relapses, with metastasis and drug resistance^5,6^. It is difficult to be treated. Ovarian cancer is one of the three major malignant tumors in the female reproductive system. Its mortality rate has always been high, posing a serious threat to women's health and lives. The studies evaluating attenuated *Salmonella typhimurium* mediated cancer treatment began in 2000. Although *Salmonella typhimurium* VNP20009 has good tumor targeting and antitumor effects in mouse tumor models, its tumor targeting ability and antitumor effect were not significant in phase I clinical trials^2^. Therefore, as a novel tumor treatment method, further research and improvement are needed for tumor-targeting bacteria. *S. typhimurium* VNP20009-Abvec-Igκ-MIIP can inhibit ovarian cancer progression by regulating Ras/MEK/ERK signaling pathway^7^. *S. typhimurium* A1-R was a deficient strain in leucine and arginine, which can widely inhibit the growth and metastasis of various tumors in mouse models, including cervical cancer, breast cancer, ovarian cancer, etc.^8^. Intraperitoneal administration of tumor-targeted *S. typhimurium* A1-R inhibited the spread of human ovarian cancer and prolonged the nude mouse survival in the experimental group^9^. *S. typhimurium* A1-R was effective for highly aggressive human ovarian cancer in metastatic and dissemination mouse models^10^. The study found that attenuated *Salmonella typhimurium* can be targeted to settle in the tumor and grow, inducing and enhancing antitumor immune cell effect, but the antitumor effect is not strong for metastatic tumors^11,12^. Bacterial L forms are cell wall-deficient bacteria. Due to bacterial L-form lack of cell wall, bacterial L forms cannot tolerate low osmotic environments and tend to survive in high osmotic pressure environments within tissues. The bacterial L forms of *S. typhimurium* are more likely to settle in the high osmotic tissues, because they are inability to tolerate low osmotic environment^13^. It is a scientific question worth discussing how effective the attenuated and bio-safety *S. typhimurium* L forms are against epithelial ovarian cancer and what the related mechanisms are. It is expected to be used for the clinical treatment of EOC.

## Materials and methods

### Bacterial strain and cell line

Bacterial strain: attenuated *Salmonella typhimurium* VNP20009 (strain YS1646) was purchased from Hangzhou Hongsai Biotechnology Co., Ltd. Cell line: The mouse ovarian cancer ID8 cell line (Cell Cook at: CC90105) was purchased from Guangzhou Saiku Biological Co., Ltd.

### Main reagents and instruments

Bacterial culture medium, cell culture medium, and serum were purchased from Jiangsu Kaiji Biotechnology Co., Ltd. CCK8 reagent and Giemsa stain were purchased from Beyotime Biotechnology Company; Rabbit anti-Mouse Lgals9 Antibody and Rabbit anti-Mouse E-cadherin Antibody were purchased from Proteintech Company; Rabbit anti-Mouse MMP9 Antibody was purchased from Affinity Biosciences Company; and Immunohistochemistry detection kits, HE stain kit, Annexin V-FITC/PI double staining cell apoptosis detection kit and TUNEL assay kits were purchased from Jiangsu Kaiji Biotechnology Co., Ltd. Transwell Chambers, Corning Company, USA. Matri Gel, BD Company. Digital pathology slide scanner (Olympus VS200, Japan). qRT-PCR detection kits were purchased from Sangon Bioengineering (Shanghai) Co., Ltd. FACS Calibur flow cytometer (BD), fluorescence quantitative PCR instrument (ABI).

### Cell culture

Mouse ovarian cancer ID8 cells were cultured in Dulbecco modified Eagle medium (DMEM; Gibco, Grand Island, NY, USA) containing 10% fetal calf serum (FCS; Gibco) at 37 °C and 5% CO_2_.

### Cell proliferation detection

Cell proliferation was analyzed using Cell Counting Kit-8 (CCK-8) assay. Cells were seeded and cultured at a density of 5 × 10^3^/well in 100 μl of medium into 96-well microplates (Corning, USA). Then, the cells were treated with the concentration of attenuated *S. typhimurium* L forms (multiplicity of infection, MOI = 10:1). Attenuated *ST* VNP20009 L forms were induced by the antibiotic ceftriaxone. Attenuated *ST* L forms become to be filamentous bodies. After treatment for 12 h, 10 μl of CCK-8 reagent was added to each well and then cultured for 3 h. All experiments were carried out in triplicate. The absorbance was analyzed at 450 nm using a microplate reader (Bio-Rad, Hercules, CA, USA) using wells without cells as blanks. The proliferation of cells was expressed by the absorbance. Cell survival activity is statistically analyzed by calculating cell viability.

### Annexin V-FITC/PI apoptosis detection

Mouse ovarian cancer ID8 cells were inoculated into a 6-well plate with 5 × 10^5^ cells per well. After the cells were attached to the wall, the medium containing *S.typhimurium* VNP20009 L forms was added with 50 μl of 10^8^ CFU/ml bacterial fluid. At the same time, negative control without *S. typhimurium* VNP20009 L forms was set, and the culture was continued for 12 h. Then, cells were prepared into single-cell suspension, stained with Annexin V/PI double staining kit, and detected by flow cytometry. The experiments were repeated at least three times. On the two-dimensional scatter plot of a bivariate flow cytometer, take the lower right quadrant (Annexin V+/PI−) to represent apoptotic cells.

### Transwell invasion experiment

For cell invasion assays, 100 μl of ID8 cells (2 × 10^5^cells/well) after attenuated *Salmonella typhimurium* L-form infection ( MOI = 10:1) or no infection was seeded with serum-free medium into the upper chamber of transwell chambers (Corning) coated with matrix gel, and 600 μl of complete medium containing 10% serum was added to the lower chamber. After incubation for 12 h at 37℃, non-invasive cells in the upper chamber were removed with a cotton swab and then fixed with 4% paraformaldehyde for 20 min and stained with 0.1% crystal violet. Counts were performed in five random fields using a microscope, and all samples were repeated three times.

### Animal test

Murine ovarian tumor assays (Animals, modeling and treatment) were conducted. The animal experiment was approved by the Animal Center of Bengbu Medical University. The approved number was No. 2022-222 of Ethical Approval for Experimental Animals at Bengbu Medical University from the Ethics Committee of Bengbu Medical University (Bengbu, China). Female C57/BL6 mice aged 6–8 weeks were derived from Henan Skbase Biotechnology Co., Ltd. Tumor models were established by subcutaneous injection of ID8 cells (5 × 10^6^/100ul). When the tumor volume reached about 100 mm^3^ after 14 days of subcutaneous injection, the mice were randomly divided into a negative control group and an attenuated *ST* VNP20009 L-form infection group. Each group of five mice had undergone three experiments. The control group was injected with 20 ul PBS every two weeks, and the experimental infection group was injected with attenuated *ST* VNP20009 L-form bacterial fluids (2 × 10^6^ CFU/ mouse) every two weeks, on the 1st, 15th, 29th, and 43rd days of the bacterial treatment experiment. The long diameter (L) and wide diameter (W) of the tumor were measured with calipers, and the tumor volume was calculated according to the formula L × (W)^2^/2. The intraperitoneal injection method of sodium pentobarbital was used to euthanize mice on day 46 of the bacterial-mediated tumor therapy experiment.

### TUNEL apoptosis detection

Tumor tissues were isolated from mice after the experimental mice were sacrificed on day 46 of the bacterial-mediated tumor therapy experiment. The tissues were immediately fixed in 4% paraformaldehyde for 24 h and embedded in paraffin. The embedded sections were sliced into 5 μm sections for staining, deparaffinized, and rehydrated for TUNEL staining following the manufacturer's instructions for the TUNEL assay kit. The results were observed using a light microscope. Image analysis was performed using Image Pro-Plus Software (Image Pro-Plus 6.0; Media Cybernetics, Silver Spring, MD, USA), and the apoptotic rate was calculated as the number of TUNEL-positive brown nuclei in DAB color rendering.

### HE staining and IHC detection

Tumor tissues were isolated from mice after the mice were sacrificed on day 46 of the bacterial-mediated tumor therapy experiment. The tissues were immediately fixed in 4% paraformaldehyde for 24 h and embedded in paraffin. The embedded sections were sliced into 5 μm sections for staining. The deparaffinized and rehydrated sections were heated in citrate buffer at 121 °C for 30 min to retrieve antigenic activity. The sections were incubated with 0.3% hydrogen peroxide in methanol for 30 min to inhibit endogenous peroxidase activity. After nonspecific reactions had been blocked with 10% normal bovine serum, the sections were incubated with rabbit polyclonal antibodies specific to Lgals9 (dilution ratio: IHC 1:200), MMP9 (dilution ratio: IHC 1:100) and E-cadherin (dilution ratio: IHC 1:5000) at 4 °C for 12 h. Then, the sections were washed with PBS and incubated with horseradish peroxidase-conjugated secondary antibody at 37 °C for 2 h. The sections were counterstained with hematoxylin for detection. Tumor tissues were fixed with 4% paraformaldehyde overnight. Subsequently, these organs were dehydrated in 25% sucrose, sectioned into 5 μm slices and stained with hematoxylin and eosin (H&E). The stained sections were imaged under an inverted phase-contrast microscope. On the basis of the number of immunohistochemical staining positive cells, the immunohistochemical scores were given. Based on widely recognized immunohistochemical analysis standards, Cells with < 10% staining were scored as negative staining (−, 1); cells with 10–49% staining were scored as (+, 2); cells with 50–74% staining were scored as (++, 3); and cells with 75–100% staining were scored as (+++, 4). The staining color was scored as none-yellow particle (0), light-yellow particle (1), brown-yellow particle (2), and brown particle (3). The final score was defined as staining number score plus staining color score. The scores of negative expression were between 0 and 2, and the scores that exceeded 2 were identified as positive expression. IHC score (+) between 2 and 3, IHC score (++) between 4 and 5, IHC score (+++) between 6 and 7.

### Fluorescence quantitative RT-PCR detection

The primers were synthesized by Sangon Bioengineering (Shanghai) Co., Ltd. The primer sequences are shown in the following description. Mouse Lgals9 Forward primer ACCTTCCAGACTCAGAACTTTCG, Reverse primer TGGACTTGGACGGGTAAAGC, product length = 190; Mouse E-cadherin Forward primer TCAACGATCCTGACCAGCAG, Reverse primer GCTGCTTGGCCTCAAAATCC, product length = 99; Mouse MMP9 Forward primer CAGCCAGACACTAAAGGCCA, Reverse primer TCATCGATCATGTCTCGCGG, product length = 153; Mouse GAPDH Forward primer GGTTGTCTCCTGCGACTTCA, Reverse primer TGGTCCAGGGTTTCTTACTCC, product length = 183.

Tumor tissues were isolated from mice after the mice were sacrificed on day 46 of the bacterial-mediated tumor therapy experiment. Tumor cells were collected. Total RNA was extracted from each group. The extracted RNA was reverse-transcribed into cDNA according to the instructions of the reverse transcription kit, and reacted in the 7300 instruments (ABI) by the way of SYBR Green I real-time RT PCR method. Primers are shown in the above description. GAPDH gene was set as internal control, and the mRNA expression levels of related genes in cells were analyzed by real-time 2^−△△^Ct method.

### Statistical analysis

Statistical data were all represented by mean ± standard deviation. The SPSS (version 26.0) statistical software was used for statistical analysis. Paired* t* tests were used to analyze the differences between the two groups. *P* < 0.05 was considered statistically significant.

### Ethics approval and consent to participate

All procedures involving animals were in compliance with the relevant guidelines and regulations of the Ethics Committee of Bengbu Medical University, and ethical approval was granted by the Ethics Committee of Bengbu Medical University (Approved number: No. 2022-222, Bengbu, China). The study is reported in accordance with ARRIVE guidelines.

## Results

### Induction of *Salmonella* typhimurium L forms

**Figure 1:**
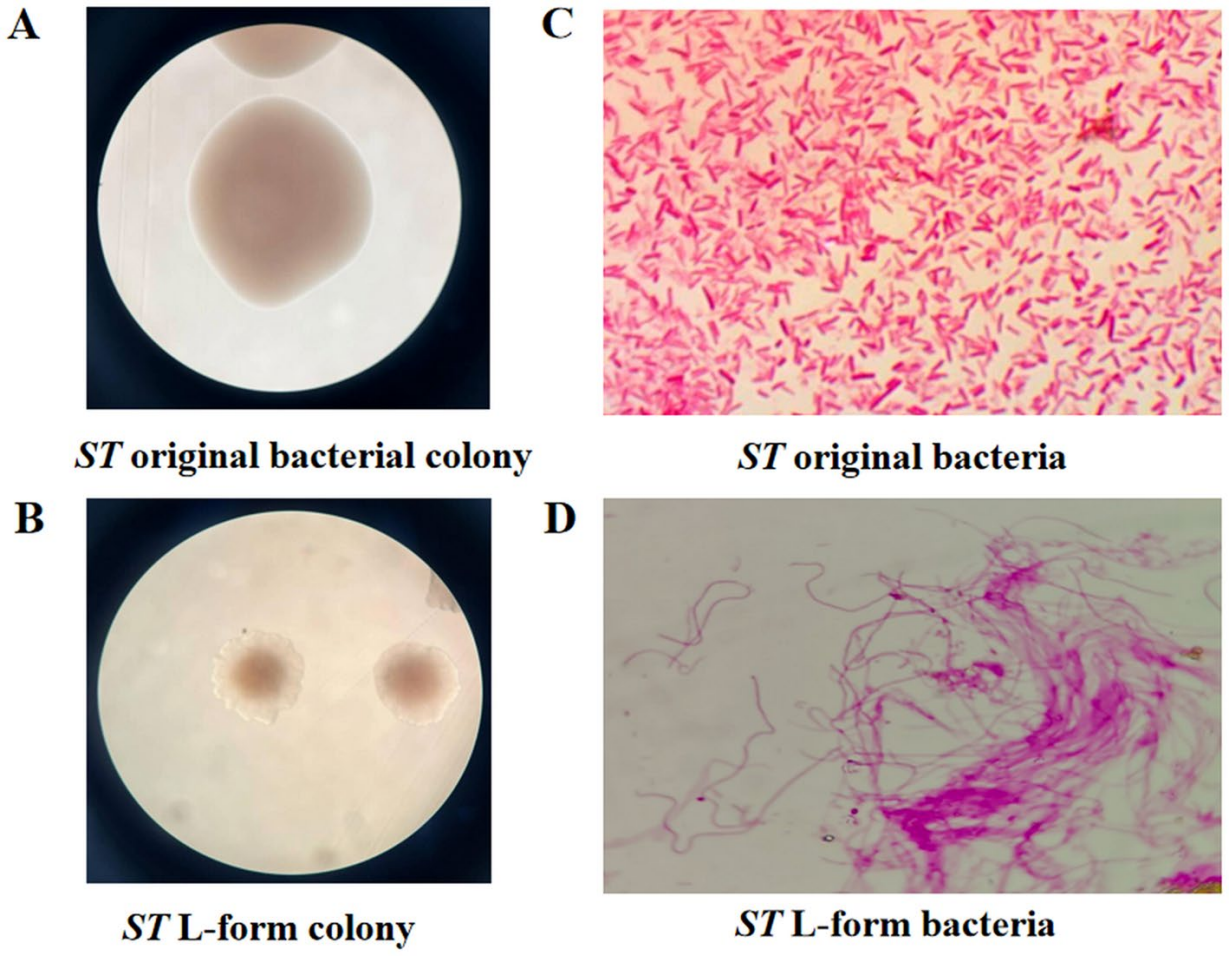
*Salmonella typhimurium* and its L forms. *Note*: (**A**) Attenuated *ST* VNP20009 strain original bacterial colony, Observing under a ×40 magnification microscope; (**B**) the L-form colonies of the attenuated *Salmonella typhimurium* VNP20009 strain induced by antibiotic ceftriaxone were observed under a ×40 magnification microscope. The L-form colonies were fried egg-like small colonies; (**C**) by Gram staining and Magnification: ×1000, *ST* original bacteria are observed to be red gram-negative bacteria, short rod-shaped, and dispersed in arrangement; (**D**) *ST* L-form bacteria are unevenly colored red gram-negative bacteria, with long filamentous bodies that intertwine into clusters, Gram staining and Magnification: ×1000.

We have successfully induced attenuated *Salmonella typhimurium* VNP20009 strain into filamentous bacterial L forms by using the antibiotic ceftriaxone sodium. By Gram staining and Magnification: 1000×, *Salmonella typhimurium* original bacteria are observed to be red gram-negative bacteria, short rod-shaped, and dispersed in arrangement. *Salmonella typhimurium* L-form bacteria are unevenly colored red gram-negative bacteria, with long filamentous bodies that intertwine into clusters, as shown in Fig. 1.

### Attenuated *ST* L forms inhibit ID8 cell proliferation and induce apoptosis

**Figure 2:**
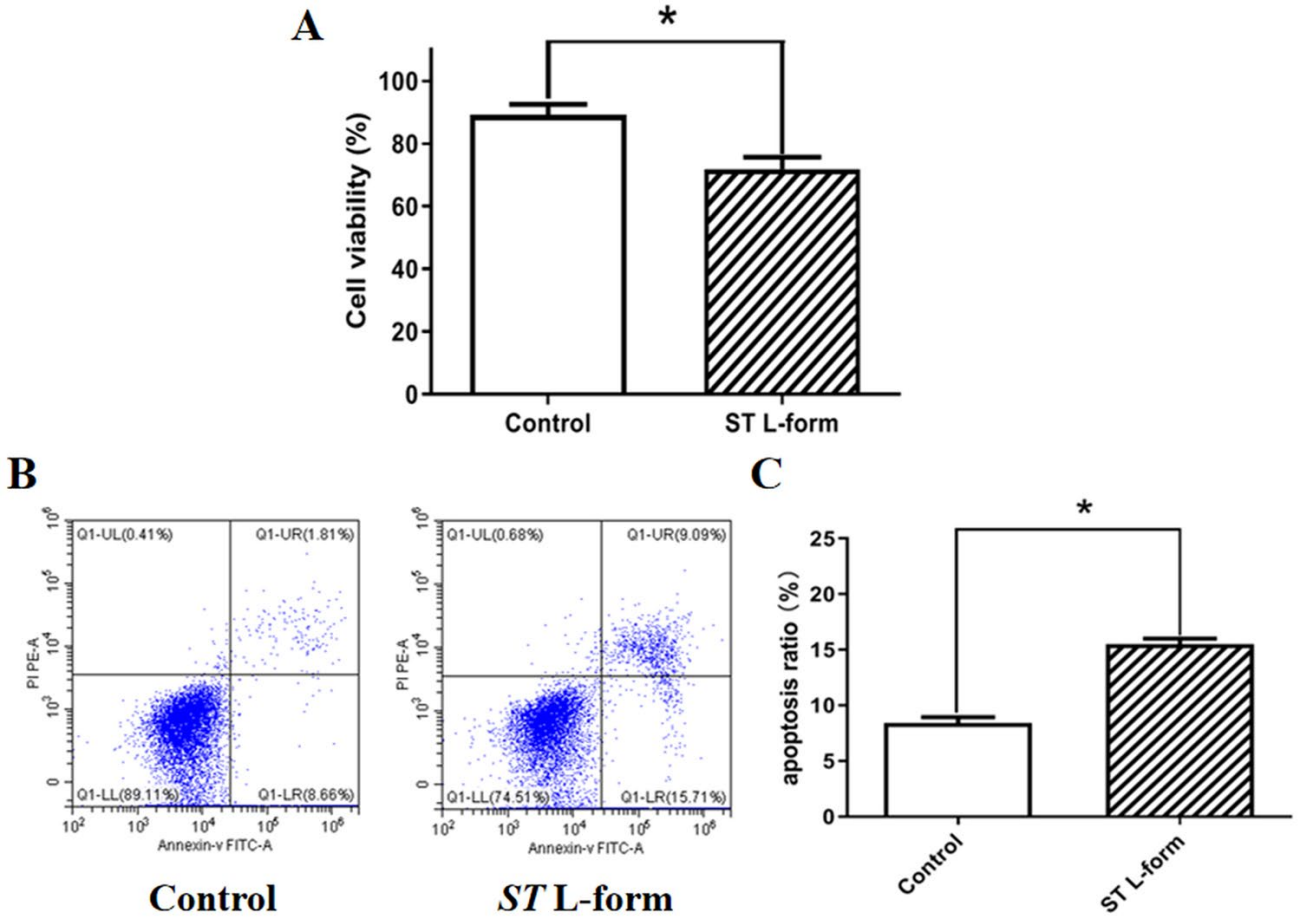
Attenuated *ST* L forms inhibit ID8 cell proliferation and induce apoptosis. *Note*: (**A**) CCK-8 assay result analysis. Attenuated *S. typhimurium* VNP20009 L-form bacteria can inhibit the growth of mouse epithelial ovarian cancer ID8 cells, *P < 0.05. (**B**) FCM apoptosis detection results. (**C**) Statistical chart analysis of apoptosis detection. Attenuated *S. typhimurium* VNP20009 L-form bacteria can induce mouse EOC ID8 cell apoptosis. *P < 0.05.

CCK8 assay was used to detect cell proliferation after co-culture 12 h of attenuated *Salmonella typhimurium* L-form bacteria and murine ovarian epithelial cancer ID8 cells (multiplicity of infection, MOI = 10:1). Attenuated *Salmonella typhimurium* VNP20009 L-form bacteria can inhibit the growth of mouse epithelial ovarian cancer id8 cells, shown in Fig. 2A. To take the lower right quadrant (Annexin V+/PI− to represent apoptotic cells, FCM apoptosis detection showed that attenuated *S. typhimurium* L forms can induce mouse ovarian cancer ID8 cell apoptosis at 12 h after their co-culture, shown in Fig. 2B,C.

### Invasiveness evaluation via Transwell invasion experiment

**Figure 3:**
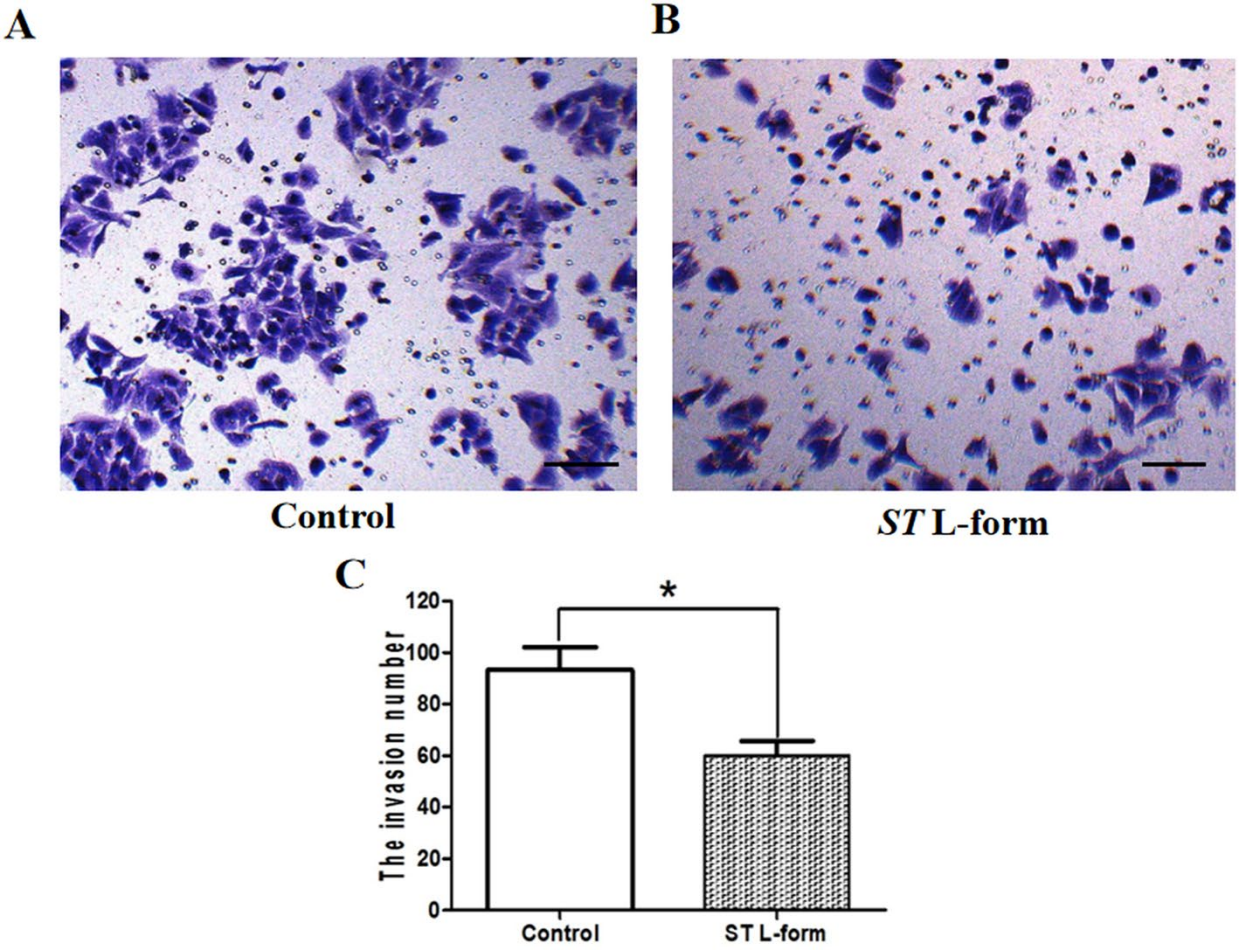
Invasiveness evaluation via transwell invasion experiment. *Note*: (**A**) Transwell invasion experiment in the blank control group of mouse EOC ID8 cells, scale bar: 50 μm; (**B**) Transwell invasion experiment was conducted in the experimental group of mouse EOC ID8 cells treated with attenuated *S. typhimurium* VNP20009 L-form bacteria, scale bar: 50 μm; (**C**) Statistical analysis of the transwell invasion experiment results, *P < 0.05.

The effect of attenuated *S. typhimurium* VNP20009 L-form bacteria on the invasion ability of mouse EOC ID8 cells was studied in the way of transwell invasion experiment of matrix gel. Attenuated *S. typhimurium* L forms can significantly reduce the invasion ability of mouse epithelial ovarian cancer ID8 cells, *P < 0.05. Observations under a high-power microscope showed a significant decrease in the number of invasive cells in the experimental group compared to the blank control group, shown in Fig. 3.

### Attenuated *ST* L forms suppress ID8 transplanted tumor growth

**Figure 4:**
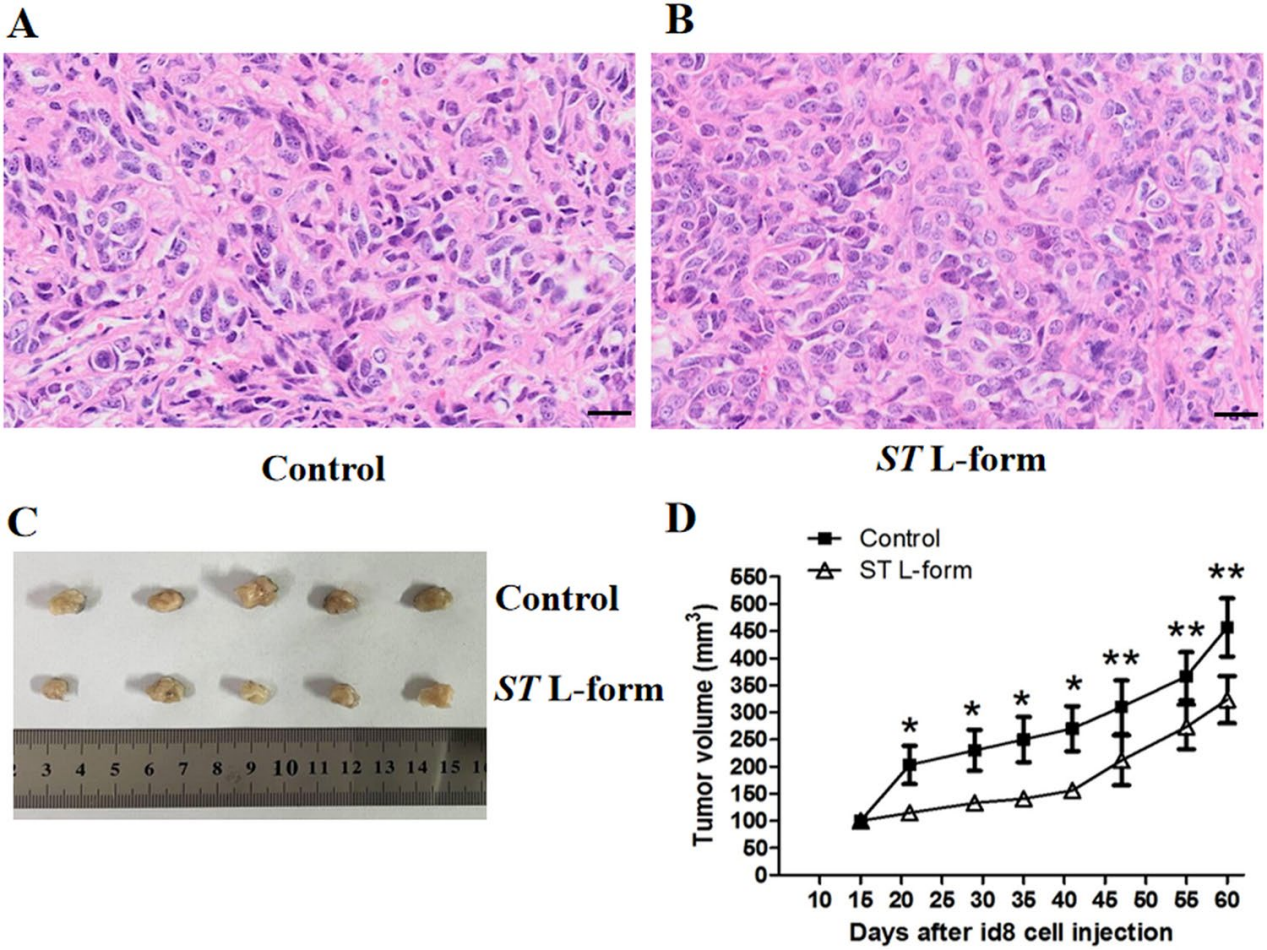
Attenuated *ST* L forms suppress ID8 transplanted tumor growth. *Note*: (**A**) HE staining of tissue sections of the transplanted tumors of mouse EOC ID8 cells in the blank control group, magnification: ×400, showed active growth of cancer cells, significant nuclear heterogeneity, and large nuclear plasma ratio, scale bar: 50 μm; (**B**) After treatment with attenuated *ST* VNP20009 L-form bacteria, HE staining was performed on the tissue sections of the transplanted tumors of mouse EOC ID8 cells, scale bar: 50 μm. When magnified 400 times and observed, they were found that the growth of cancer cells was not active; (**C**) 60 days after transplantation of mouse epithelial ovarian cancer ID8 cell line, the actual tumor image; (**D**) Statistical analysis of the experimental results of two groups of tumor growth volume detection, *P < 0.05, **P < 0.01.

We studied the effect of *ST* L forms mediated EOC therapy. The effects of attenuated *ST* VNP20009 L-form bacteria on the transplanted tumor growth of mouse EOC ID8 cells were observed day by day from the bacterial treatment experiment beginning. Treatment with attenuated *ST* VNP20009 L-form bacteria can lead to a slowdown in the transplanted tumor growth and volumes of mouse EOC ID8 cells, shown in Fig. 4. Tumour tissues were isolated from mice after mice were sacrificed on day 46 of the bacterial-mediated tumor therapy experiment. The tissues were immediately fixed in 4% paraformaldehyde for 24 h and embedded in paraffin. Then we use the embedded tissue pathological sections for HE staining and IHC detection.

### Attenuated *ST* L forms promote apoptosis in murine ovarian tumors

**Figure 5:**
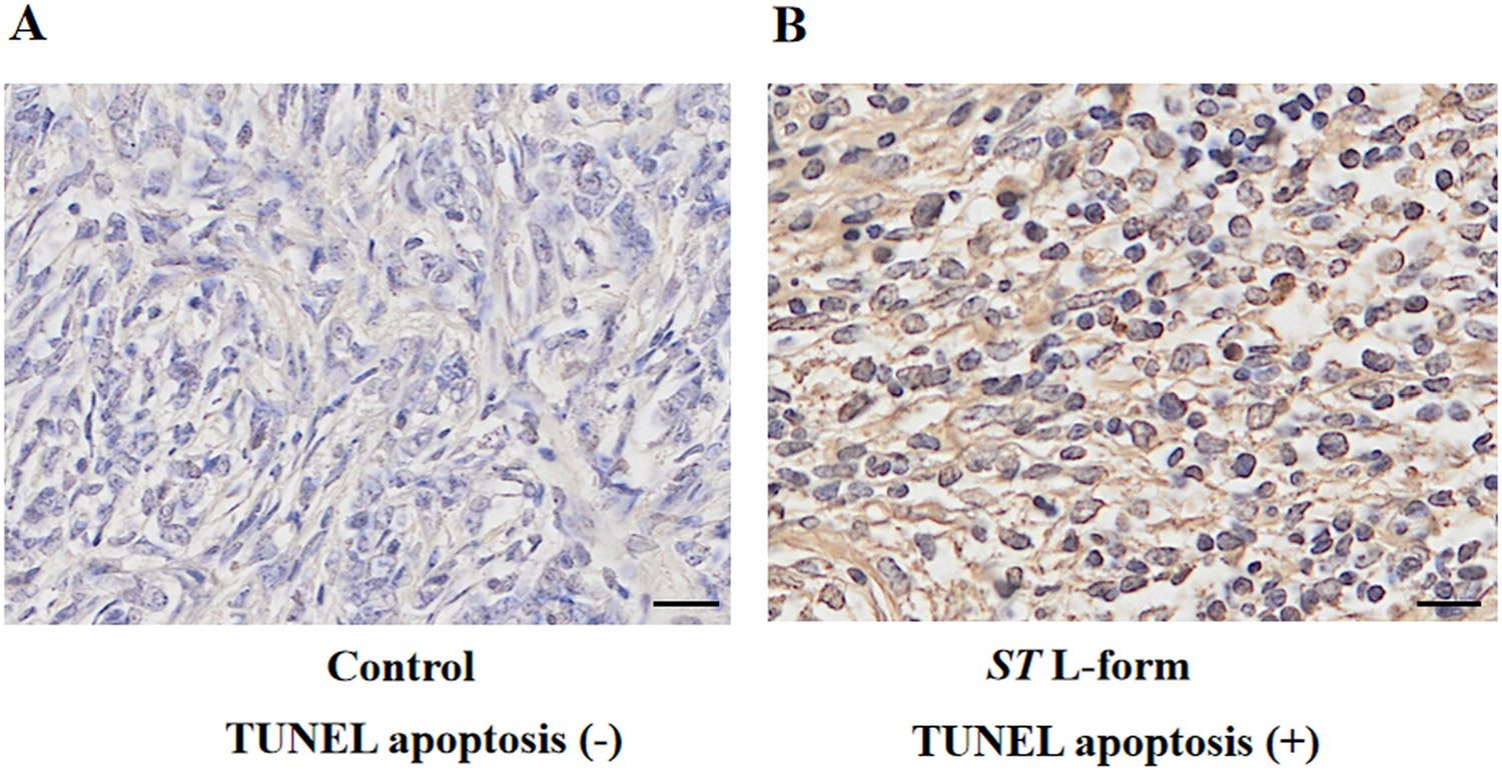
Attenuated *ST* L forms promote apoptosis in murine ovarian tumors. *Note*: (**A**) TUNEL apoptosis detection in the blank control group showed that apoptosis in the tumors of mouse EOC ID8 cell line was very rare, scale bar: 50 μm; (**B**) After treatment with attenuated *S. typhimurium* VNP20009 L-form bacteria, there was significant apoptosis in the tumors of mouse EOC ID8 cells, scale bar: 50 μm. TUNEL-positive apoptosis cell nuclei of DAB color rendering were brown. Attenuated *S. typhimurium* VNP20009 L-form bacteria can induce cancer cell apoptosis in murine ovarian tumors.

The effect of attenuated *ST* VNP20009 L-form bacteria on apoptosis of mouse EOC ID8 transplanted tumor cells was studied in murine ovarian tumors. Tumor tissues were isolated from mice after mice were sacrificed on day 46 of the bacterial-mediated tumor therapy experiment. TUNEL apoptosis detection showed that attenuated *ST* L forms can induce apoptosis of mouse EOC ID8 transplanted tumor cells. The TUNEL-positive apoptosis cell nuclei of DAB color rendering were brown, as shown in Fig. 5.

### Effects of attenuated *ST* L-form on the expression of Lgals9, MMP9 and E-cadherin in ID8 transplanted tumors

**Figure 6:**
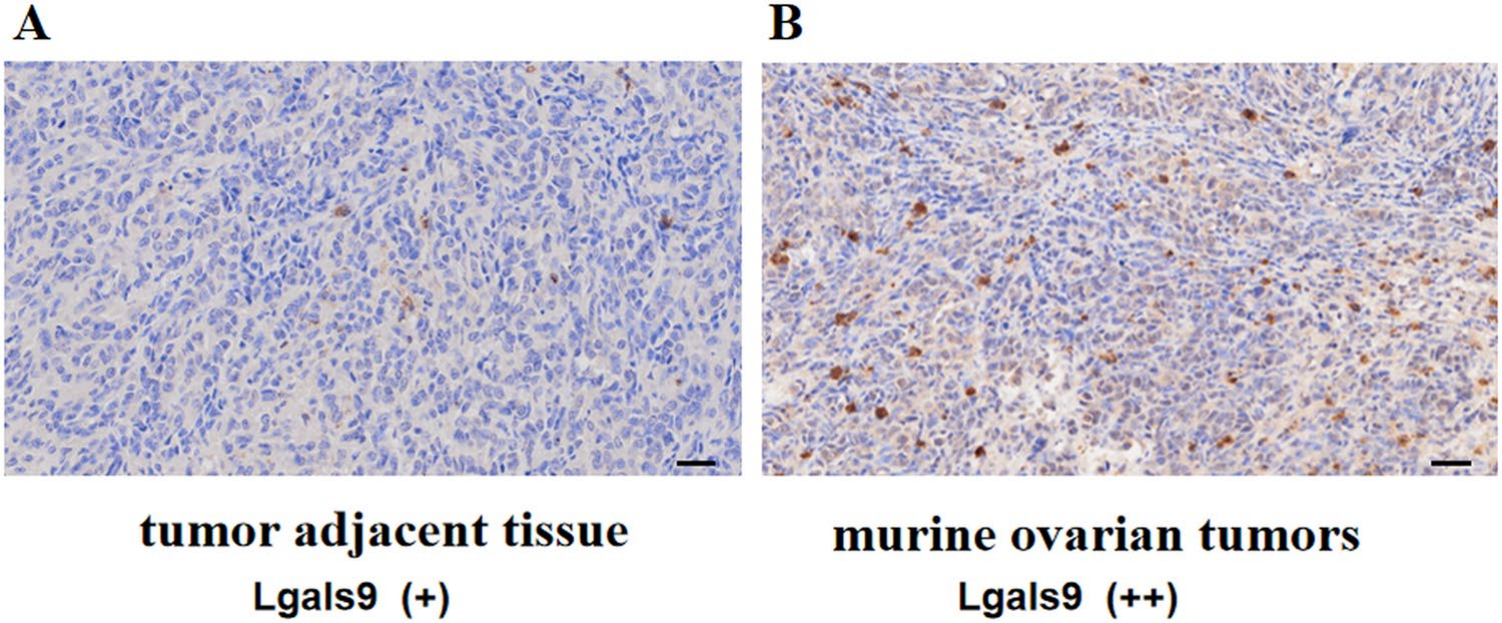
Expression of Lgals9 in the transplanted tumors of mouse EOC ID8 cells. *Note*: (**A**) The tissue adjacent to the transplanted tumors of mouse EOC ID8 cells was the low expression of Lgals9, IHC score (+), scale bar: 50 μm; (**B**) Lgals9 was highly expressed in the transplanted tumors of mouse EOC id8 cells, IHC score (++), scale scale bar: 50 μm.

**Figure 7:**
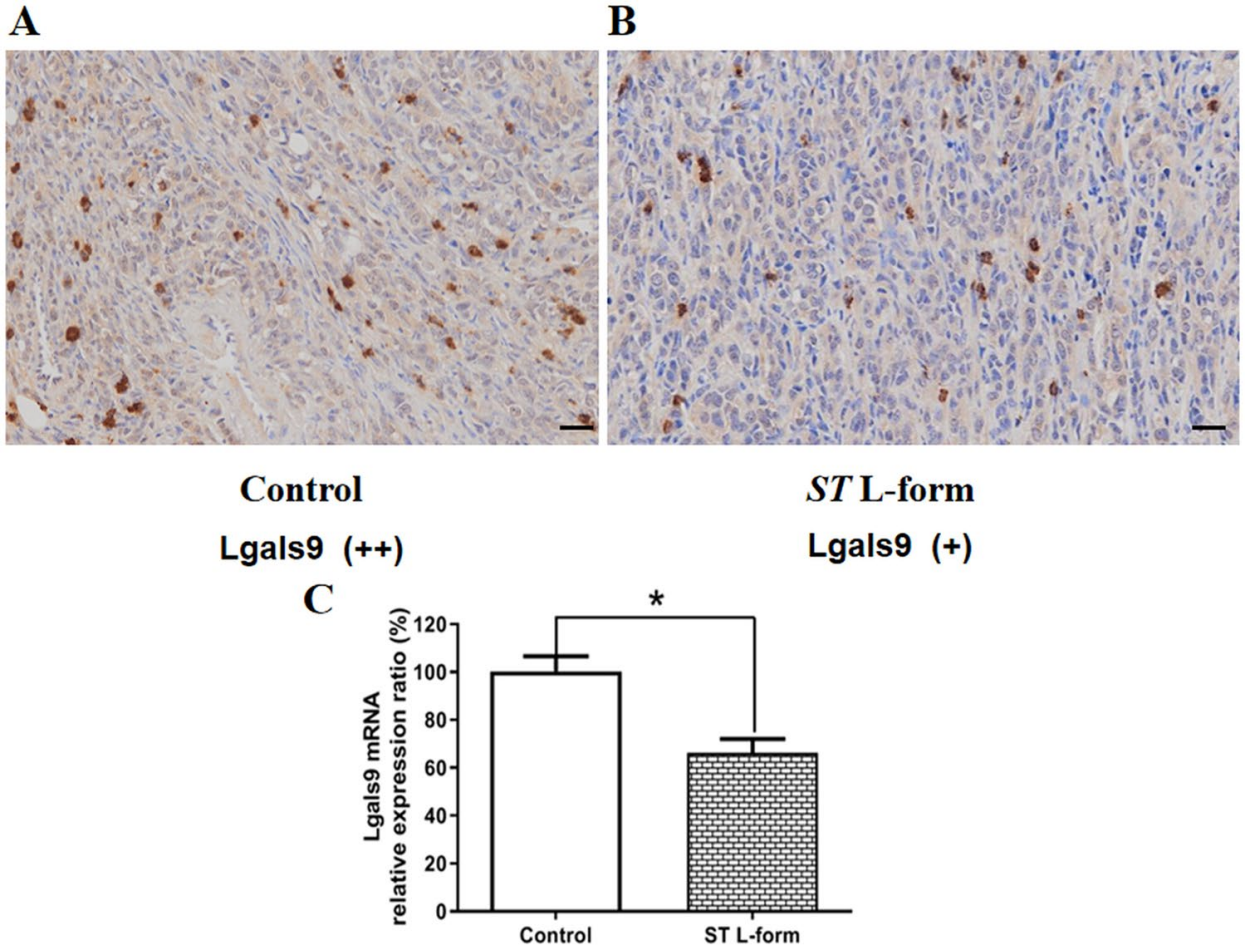
Effect of attenuated *ST* VNP20009 L forms on the expression of Lgals9 in transplanted tumors of mouse EOC ID8 cells. *Note*: (**A**) High expression of Lgals9 in transplanted tumors of mouse EOC ID8 cells, IHC score (++), scale bar: 50 μm; (**B**) After treatment with attenuated *ST* VNP20009 L-form bacteria, the expression of Lgals9 in the transplanted tumors of mouse EOC ID8 cells decreased, IHC score (+), scale bar: 50 μm; (**C**) qRT-PCR detection showed that attenuated *ST* L forms inhibited the expression of Lgals9 mRNA in mouse ID8 transplanted tumors (*P < 0.05).

**Figure 8:**
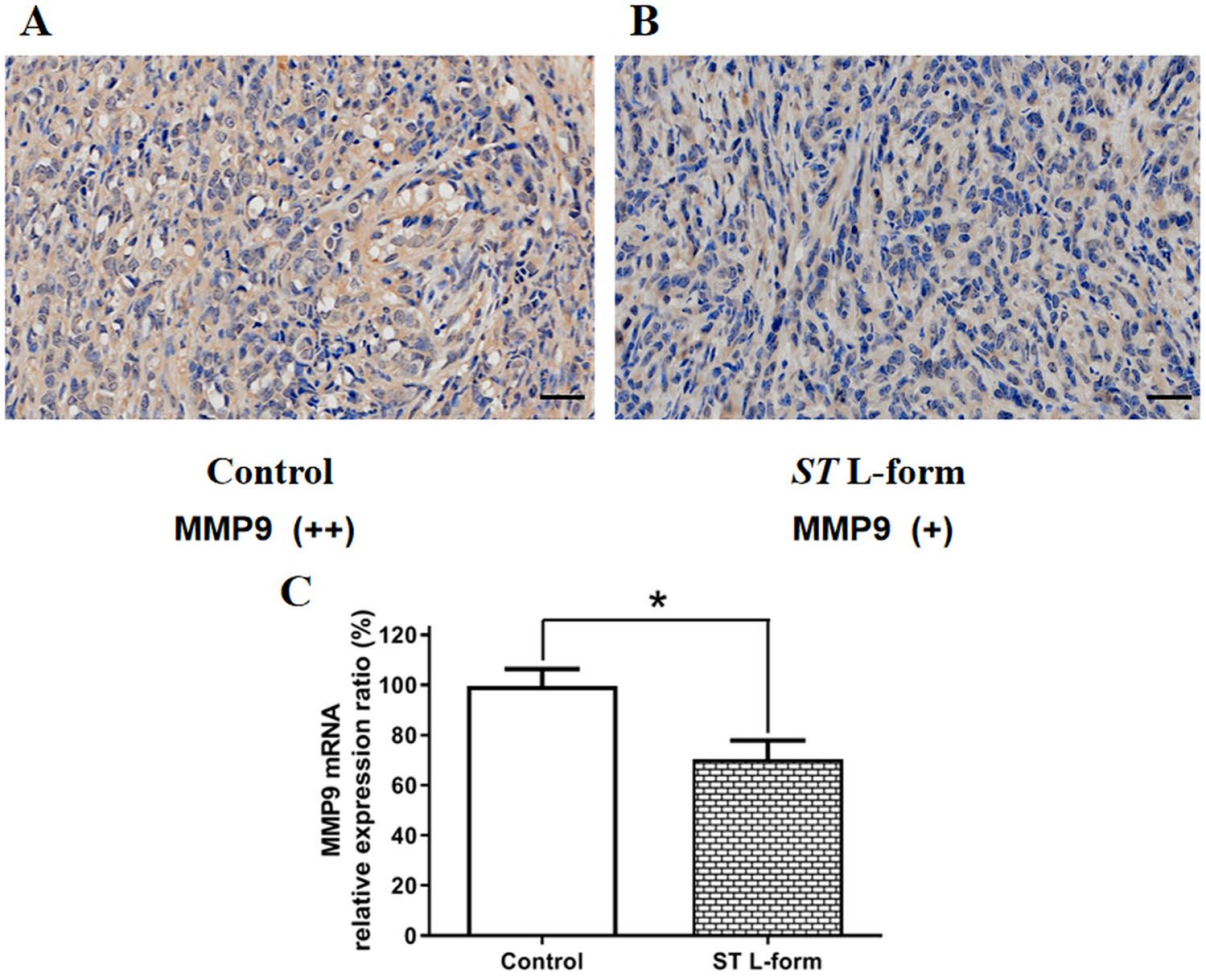
Effect of attenuated *ST* L forms on the expression of MMP9 in mouse EOC ID8 tumors. *Note*: (**A**) High expression of MMP9 in transplanted tumors of mouse EOC ID8 cells, IHC score (++), scale bar: 50 μm; (**B**) After treatment with attenuated *ST* VNP20009 L-form bacteria, the expression of MMP9 in the transplanted tumors of mouse EOC ID8 cells decreased, IHC score (+), scale bar: 50 μm; (**C**) qRT-PCR detection showed that attenuated *ST* L forms inhibited the expression of MMP9 mRNA in mouse ID8 transplanted tumors (*P < 0.05).

**Figure 9:**
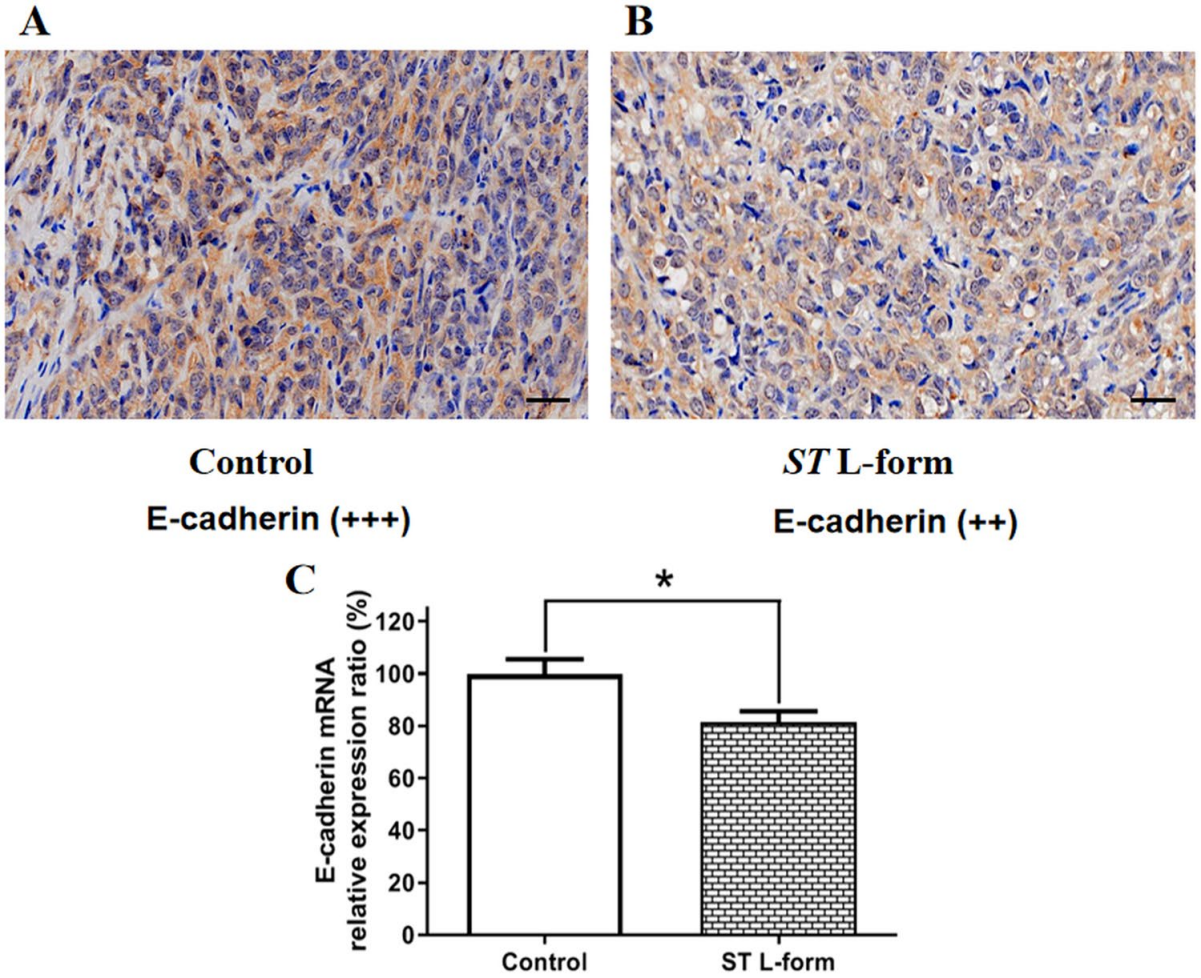
Effect of attenuated *ST* L forms on the expression of E-cadherin in mouse EOC ID8 tumors. *Note*: (**A**) High expression of E-cadherin in transplanted tumors of mouse EOC ID8 cells, immunohistochemical score (+++), scale bar: 50 μm; (**B**) After treatment with attenuated *ST* VNP20009 L-form bacteria, the expression of E-cadherin in the transplanted tumors of mouse EOC ID8 cells decreased, IHC score (++), scale bar: 50 μm; (**C**) qRT-PCR detection showed that attenuated *ST* L forms inhibited the expression of E-cadherin mRNA in mouse ID8 transplanted tumors (*P < 0.05).

Tumorigenicity-related proteins of xenograft tumors were detected by immunohistochemistry assays, such as Lgals9, MMP9 and E-cadherin. The effects of attenuated *ST* VNP20009 L-form bacteria on the expression of Lgals9 protein in transplanted tumors of mouse EOC ID8 cell line were investigated. The tissue adjacent to the transplanted tumors of mouse EOC ID8 cells was the low expression of Lgals9, but Lgals9 was highly expressed in the transplanted tumors of mouse EOC ID8 cells, shown in Fig. 6. After treatment with attenuated *ST* VNP20009 L-form bacteria, the expression of Lgals9 in the transplanted tumors of mouse EOC ID8 cells decreased, shown in Fig. 7. The low expression of Lgals9 can enhance the antitumor immune function of the mouse body. Attenuated *ST* L forms can reduce the expression of proteins and mRNA that promote tumor growth and metastasis, such as Lgals9 and MMP9, shown in Figs. 7 and 8. In addition, we also found that attenuated *ST* L forms can reduce the expression of E-cadherin proteins and mRNA in the transplanted tumors of mouse EOC ID8 cells, shown in Fig. 9.

## Discussion

In recent years, the incidence and mortality of cancer have remained high throughout the world. Surgical treatment, chemotherapy, Radiotherapy and immunotherapy are still the main methods of tumor treatment, and the targeting and universality of tumor treatment need to be improved. In the middle and late stages of cancer, metastatic cancer cells break away from the primary tumor, infiltrate surrounding tissues, spread to the whole body, and form metastatic lesions in different organs and tissues of the human body, which has become the main cause of death in cancer patients. Among them, epithelial ovarian cancer (EOC) accounts for about 90% of the pathological types of ovarian cancer^14^. Due to the concealed anatomical location of ovarian cancer, it is clinically found that ovarian cancer is mostly in the middle and late stages of tumor development, and epithelial ovarian cancer has spread directly to the abdominal cavity for metastasis. After surgery combined with platinum-based chemotherapy, patients are still prone to metastasis, recurrence, and drug resistance, resulting in a low 5-year survival rate. Ovarian cancer (OC) is the main cause of death in gynecological malignancies. Therefore, there is a need to find new therapeutic approaches to improve treatment outcomes for patients with ovarian cancer. Our laboratory further explored the antitumor effect of attenuated *Salmonella typhimurium* VNP20009 L forms, which may bring hope for the treatment of patients with epithelial ovarian cancer.

Bacterial-mediated tumor therapy has become a hot topic in the field of antitumor therapy. *Salmonella typhimurium* can specifically colonize and proliferate in tumors and inhibit tumor growth. It is one of the main bacterial strains for bacterial therapy^15^. Among them, attenuated *S. typhimurium* VNP20009 is commonly used in cancer-targeted therapy and synergistically regulates the tumor microenvironment by combining with radiotherapy, chemotherapy, and other therapeutic methods^16^. The attenuated *S. typhimurium* VNP20009 is a patent strain, a genetically modified strain, that partially deleted the msbB gene and the purI gene encoding lipid A and adenine on the basis of *Salmonella typhimurium* ATCC14028 strain, reducing toxicity^2,16^. The mutant strain of *Salmonella typhimurium* VNP20009 has a deficiency in the purI gene, which prevents it from synthesizing adenine. The required adenine can only be obtained from external sources, and a large amount of adenine is present in tumor cells. Therefore, *Salmonella typhimurium* VNP20009 prefers to settle inside tumors. The deletion of the msbB gene causes certain changes in the structure of bacterial lipid A, thereby reducing the virulence of the strain and reducing the likelihood of inducing septic shock in the body during administered systemic.

Attenuated *Salmonella typhimurium* L forms lack complete cell walls, so as to be weakened in the ability to resist osmotic stress^17^. Compared with the original bacteria, bacterial L forms are more likely to colonize deep in the host tumor tissues and cause interstitial inflammation, thus playing an antitumor role. In our research, attenuated *Salmonella typhimurium* VNP20009 L forms were induced by using antibiotic ceftriaxone. Evaluation of their anti-epithelial ovarian cancer efficacy and the related mechanisms were studied, by means of in vitro cell experiments and in vivo animal experiments, and it was prospected for the future treatment of epithelial ovarian cancer. Our research is different from previous antitumor studies using attenuated *Salmonella typhimurium*, and has a certain degree of innovation and exploration.

We investigated whether attenuated *Salmonella typhimurium* L forms can decrease the growth and invasion of ovarian epithelial cancer cells. The roles of Lgals9, MMP9, and E-cadherin in epithelial ovarian cancer were investigated by immunohistochemical assays and qRT-PCR detection.

Lgals9 (Galactose lectin 9), a member of the galactose lectin family, is a widely expressed protein that is involved in immunomodulation and tumor pathogenesis and influences the prognosis of many types of cancers^18^. Lgals9 is a ligand for TIM3, and the Lgals9/TIM3 pathway has immunomodulatory effects such as regulating apoptosis of T helper cells, depletion of CD8^+^ T cells, activation of CD4^+^ T cells and NK cells^19^. In the tissues surrounding the tumors transplanted with mouse EOC ID8 cells, Lgals9 expression was low, but Lgals9 expression was high in transplanted tumors. The expression of Lgals9 in the transplanted tumors of mouse EOC ID8 cells was decreased after treatment with attenuated *Salmonella typhimurium* VNP20009 bacterial L forms, and the antitumor immune function of the mouse organism might been improved. Because of binding to its receptor TIM-3, Lgals9 can inhibit Th1 and Th17 amplification, promote CD8^+^ T cell depletion, and inhibit the body's antitumor immunity^19^.

Matrix metalloproteinase-9 (MMP-9) is one of the most complex forms of matrix metalloproteinases^20^. MMP9 is an enzyme that mainly degrades type IV collagen and elastin. On the one hand, it promotes tumor cell growth by promoting the generation of new blood vessels, and on the other hand, it promotes tumor cell infiltration and invasion into the basement membrane by degrading and destroying the basement membrane^21,22^. MMP9 is significantly elevated in tissues of various malignant solid tumors, becoming a target for antitumor drugs. Immunohistochemistry assays revealed a decrease in MMP9 expression in tumor tissue from mouse EOC ID8 cells after treatment with attenuated *ST* VNP20009 bacterial L forms, indicating that attenuated *ST* L-form treatment is useful in treating epithelial ovarian cancer and can be used as a potent anticancer drug for future research.

An essential component for preserving the integrity and polarity of epithelial cells is E-cadherin, an intercellular adhesion molecule that primarily mediates the adhesion reaction between homologous cells^23,24^. Tumor metastasis may be encouraged by dysregulated E-cadherin expression, which can also reduce intercellular adhesion^25,26^. In this experiment, the expression of E-cadherin in tumor tissues was elevated. After treatment with attenuated *ST* VNP20009 bacterial L forms, E-cadherin expression in transplanted tumors of mouse EOC ID8 cells was decreased, resulting in reduced adhesion between cancer cells. When the tumor is too small in volume, the low expression of E-cadherin will be detrimental to cancer cell proliferation and tumor growth.

In the realm of antitumor therapy, bacteria-mediated tumor therapy has emerged as a hotspot for research^27^. In particular, *Salmonella typhimurium* has the ability to colonize, multiply, and stop the growth of malignancies. It is a primary strain used in bacterial treatment. *Salmonella typhimurium* is more likely than other bacteria to aggregate in the spaces between cancer cells in malignant tumor tissues, triggering antitumor immunity and preventing tumor growth and metastasis, according to recent studies. The modified attenuated *Salmonella typhimurium* is one of the candidates for low-cost, high-quality antitumor biological agents. It can be used to deliver antitumor drug molecules in cancer treatment, which can be better utilized to inhibit tumor development and spread. The clinical effect has to be further refined and optimized because it is now not optimal^28–30^.

Wild-type *Salmonella typhimurium* can be used as a carrier of anticancer proteins and molecules for tumor treatment, but the wild-type strain has strong biological toxicity, such as bacterial endotoxin lipopolysaccharide LPS on the bacterial cell walls, which can induce the production of inflammatory cytokines such as TNF-α. Excessive TNF-α can easily lead to septic shock, so it has not been used in clinical practice. Since then, researchers have used genetic engineering technology to modify wild-type *Salmonella typhimurium* and developed a series of attenuated mutant strains for antitumor bacterial therapy^31,32^. Attenuated *S. typhimurium* VNP20009 is a genetically modified strain without strong biological toxicity. Attenuated *Salmonella typhimurium* L forms are less resistant to low osmotic stress because of lacking complete cell walls. Bacterial L forms have a higher propensity than the original bacteria to infiltrate deeply into the host tissue, resulting in interstitial inflammation and subsequent antitumor actions. *S. typhimurium* L forms can penetrate deep into the interior of tumors and perform their growth and proliferation, thereby inhibiting the growth of ovarian cancer, plundering the nutrients inside the tumor, producing toxic metabolic waste, inducing cancer cell death, leading to restricted growth of ovarian cancer, delayed cell proliferation and tomur volume growth, and reduced expression of Lgals9, MMP9, and E-cadherin proteins in ovarian cancer cells. We found that attenuated *S. typhimurium* VNP20009 L forms can impact on tumorigenicity and induce apoptosis of mouse ovarian cancer epithelial cells in vivo (Supplementary Figures 1–5).

## Conclusion

Attenuated *Salmonella typhimurium* L forms can inhibit the proliferation and invasion ability of epithelial ovarian cancer cells, and induce apoptosis in vitro. Attenuated *ST* L forms can inhibit tumor growth and promote apoptosis in murine ovarian tumors. Attenuated *ST* L forms may serve as a novel biological agent for bacterial-mediated tumor therapy in ovarian epithelial cancer.

### Supplementary Information

Supplementary Figures.

## Data Availability

The datasets used and/or analyzed during the current study available from the corresponding author on reasonable request.
