# Supplementary material for: Attenuated Salmonella typhimurium L forms suppress tumor growth and promote apoptosis in murine ovarian tumors
Source: Sci Rep. 2024 Jul 11;14:16045. doi: 10.1038/s41598-024-66898-x (PMC11239651; doi:10.1038/s41598-024-66898-x)
Supplement: Supplementary file 1 — Supplementary Figures. [file 41598_2024_66898_MOESM1_ESM.docx]

## **Supplementary Information**

Additional file Information

# Additional file 1 Figures


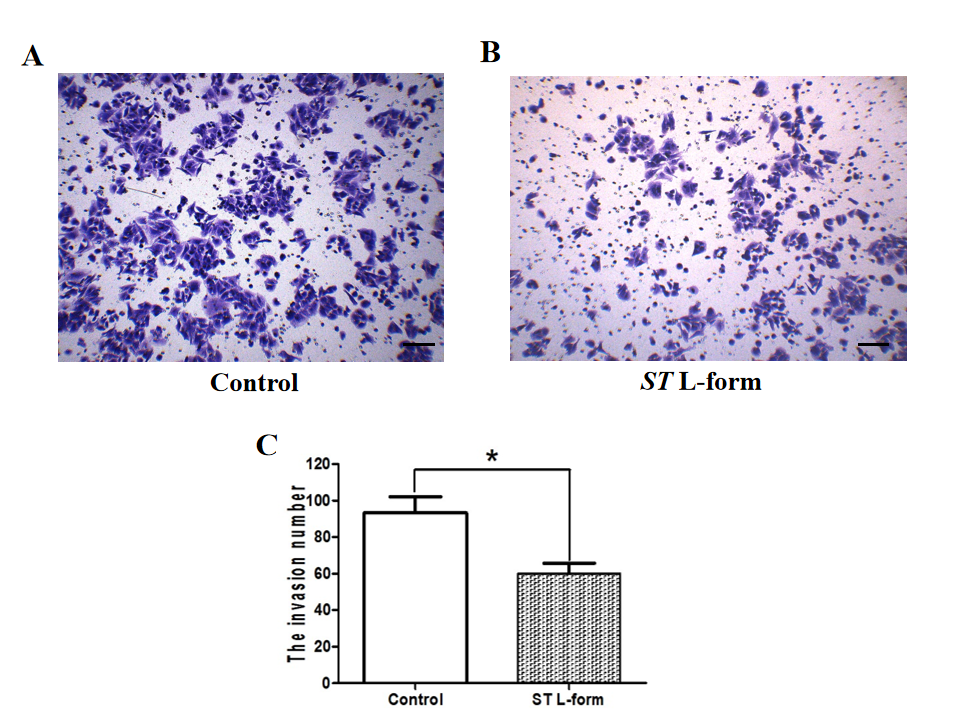


**Additional file Figure 1. Full camera view photos of transwell invasion experiment**

Note: A. Transwell invasion experiment in the blank control group of mouse EOC id8 cells, scale bar: 50μm; B. Transwell invasion experiment was conducted in the experimental group of mouse EOC id8 cells treated with attenuated *S. typhimurium* VNP20009 L-form bacteria, scale bar: 50μm.


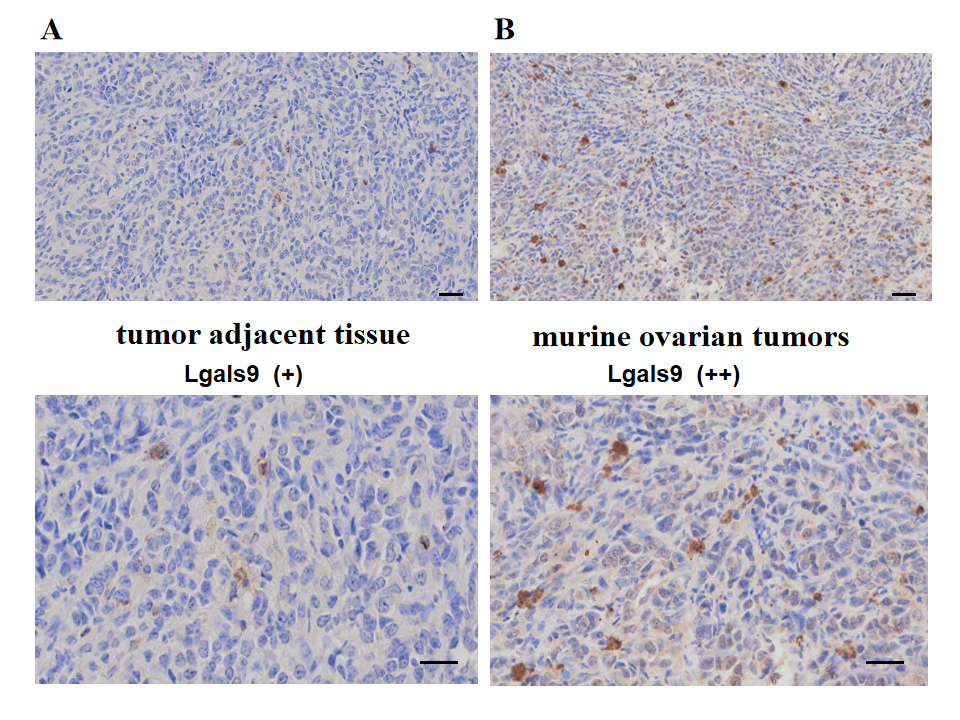


**Additional file Figure 2. Expression of Lgals9 in the transplanted tumors of mouse EOC id8 cells**

Note: A. The tissue adjacent to the transplanted tumors of mouse EOC id8 cells was the low expression of Lgals9, IHC score (+), scale bar: 50μm; B. Lgals9 was highly expressed in the transplanted tumors of mouse EOC id8 cells, IHC score (++), scale bar: 50μm.The upper image shows a large field of view, while the lower image shows a small field of view observation.

**
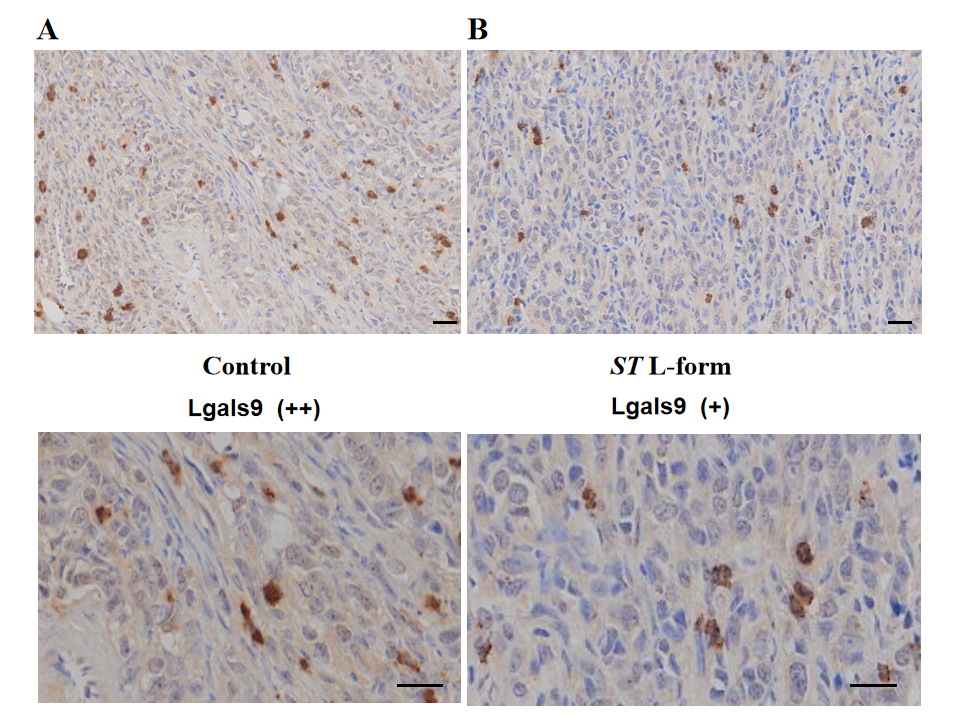
**

**Additional file Figure 3. Effect of attenuated *ST* VNP20009 L forms on the expression of Lgals9 in transplanted tumors of mouse EOC id8 cells**

Note: A. High expression of Lgals9 in transplanted tumors of mouse EOC id8 cells, IHC score (++), scale bar: 50μm; B. After treatment with attenuated *ST* VNP20009 L-form bacteria, the expression of Lgals9 in the transplanted tumors of mouse EOC id8 cells decreased, IHC score (+), scale bar: 50μm.The upper image shows a large field of view, while the lower image shows a small field of view observation.

**
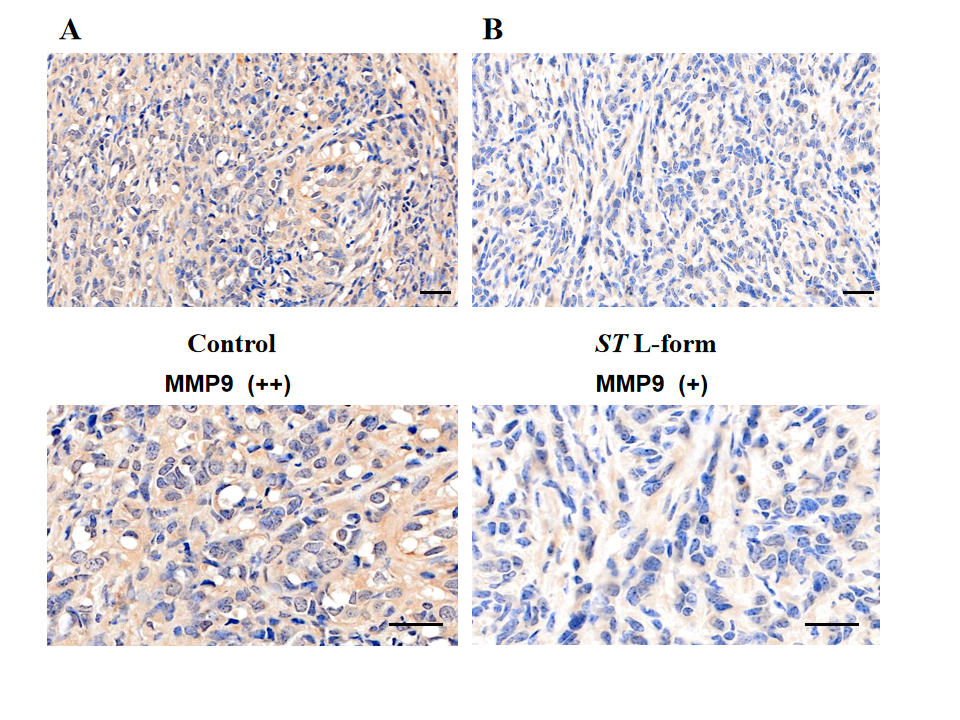
**

**Additional file Figure 4. Effect of attenuated *ST* L forms on the expression of MMP9 in mouse EOC id8 tumors**

Note: A. High expression of MMP9 in transplanted tumors of mouse EOC id8 cells, IHC score (++), scale bar: 50μm; B. After treatment with attenuated *ST* VNP20009 L-form bacteria, the expression of MMP9 in the transplanted tumors of mouse EOC id8 cells decreased, IHC score (+), scale bar: 50μm. The upper image shows a large field of view, while the lower image shows a small field of view observation.

**
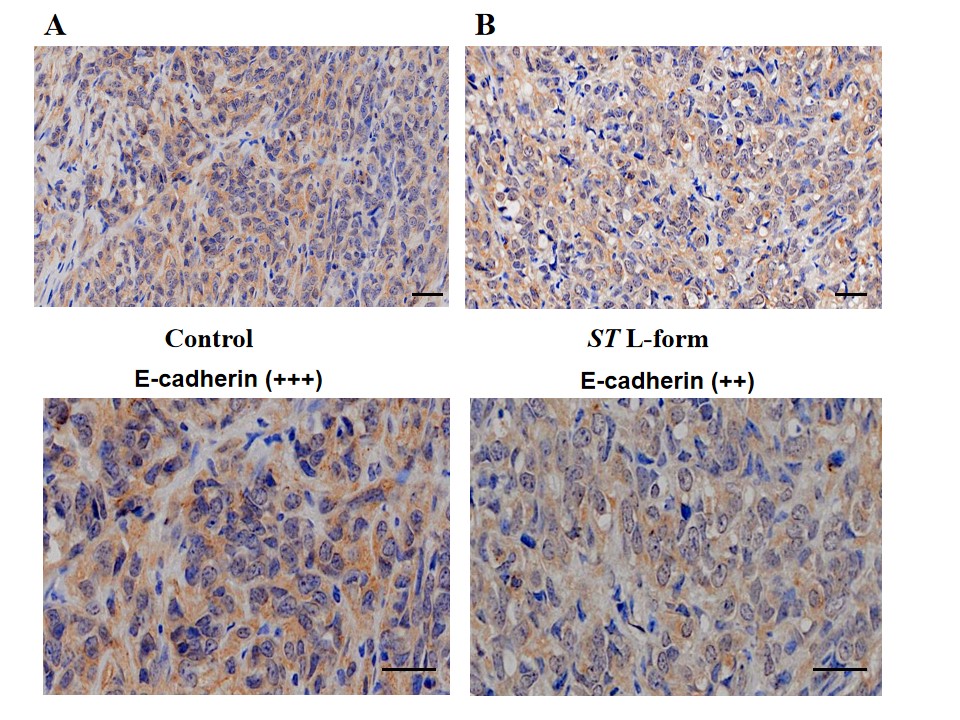
**

**Additional file Figure 5. Effect of attenuated *ST* L forms on the expression of E-cadherin in mouse EOC id8 tumors**

Note: A. High expression of E-cadherin in transplanted tumors of mouse EOC id8 cells, immunohistochemical score (+++), scale bar: 50μm; B. After treatment with attenuated *ST* VNP20009 L-form bacteria, the expression of E-cadherin in the transplanted tumors of mouse EOC id8 cells decreased, IHC score (++), scale bar: 50μm. The upper image shows a large field of view, while the lower image shows a small field of view observation.
